# Advancements in cache management: a review of machine learning innovations for enhanced performance and security

**DOI:** 10.3389/frai.2025.1441250

**Published:** 2025-02-25

**Authors:** Keshav Krishna

**Affiliations:** Department of Computer Science & Engineering, Indian Institute of Technology Ropar, Rupnagar, India

**Keywords:** cache management, cache security, cache replacement, edge networks, hardware, machine learning, reinforcement learning, deep learning

## Abstract

Machine learning techniques have emerged as a promising tool for efficient cache management, helping optimize cache performance and fortify against security threats. The range of machine learning is vast, from reinforcement learning-based cache replacement policies to Long Short-Term Memory (LSTM) models predicting content characteristics for caching decisions. Diverse techniques such as imitation learning, reinforcement learning, and neural networks are extensively useful in cache-based attack detection, dynamic cache management, and content caching in edge networks. The versatility of machine learning techniques enables them to tackle various cache management challenges, from adapting to workload characteristics to improving cache hit rates in content delivery networks. A comprehensive review of various machine learning approaches for cache management is presented, which helps the community learn how machine learning is used to solve practical challenges in cache management. It includes reinforcement learning, deep learning, and imitation learning-driven cache replacement in hardware caches. Information on content caching strategies and dynamic cache management using various machine learning techniques in cloud and edge computing environments is also presented. Machine learning-driven methods to mitigate security threats in cache management have also been discussed.

## 1 Introduction

Caching is a crucial technology in computer systems, enhancing performance by storing frequently accessed data to reduce latency. Efficient cache management involves strategic data storage, reallocation, and eviction, especially in environments like cloud computing where resources are shared among multiple tenants with varying quality of service requirements, to improve system performance (Choi et al., 2020; Sethumurugan et al., 2021). Implementing machine learning into cache management has revolutionized traditional approaches, providing dynamic, adaptive solutions that address complex, variable workloads and security concerns in modern computing environments.

Despite the pivotal role of caching in system performance and resource utilization, existing cache management policies face significant challenges. These include adapting to dynamic content popularity, efficiently utilizing limited cache spaces, and ensuring security against sophisticated attacks such as like cache side-channel exploits with low overhead, high accuracy, and adaptability (Sethumurugan et al., 2021; Vietri et al., 2018; Narayanan et al., 2018; Liu et al., 2020; Li et al., 2019; Ma et al., 2018; Tong et al., 2020). Machine learning offers innovative solutions to these issues, with techniques ranging from deep learning for predictive caching to reinforcement learning for policy optimization, presenting a transformative potential for cache management strategies.

In recent years, there has been substantial progress in both traditional and machine learning-based cache management techniques. However, most existing research has focused primarily on performance improvements without adequately addressing the dual need for enhanced security in cache management systems. Moreover, there is a lack of comprehensive studies reviewing the integration of machine learning innovations specifically aimed at optimizing both cache performance and security. This study aims to fill this gap by providing a detailed review of recent advancements in machine learning applications for cache management, emphasizing both performance and security enhancements.

This review aims to explore the breadth of recent research in machine learning-driven cache management, focusing on examining the effectiveness of various machine learning techniques, including reinforcement learning, deep learning, and imitation learning, in developing cache replacement policies, analyzing machine learning's role in enhancing cache-related security measures against evolving threats, and investigating the application of machine learning in different caching environments from edge devices to large-scale cloud systems. To the knowledge of the author, no similar review exists in the current literature covering the breadth of machine learning in cache management.

The scope of this review is confined to the study of machine learning applications in cache management. It assesses the performance enhancements these techniques offer over conventional strategies and explores their effectiveness in improving system performance and mitigating security risks in diverse computing environments.

The major contributions of the study are

- Explore the breadth of recent research in machine learning-driven cache management.
- Examine the effectiveness of various machine learning techniques in developing cache management policies: reinforcement learning, deep learning, and imitation learning.
- Analyze machine learning's role in enhancing cache-related security measures against evolving threats.
- Investigate the application of machine learning in different caching environments: hardware, edge devices and large-scale cloud systems.

The structure of this review is organized as follows: Section 1 provides an introduction to the study; Section 2 outlines a detailed examination of machine learning applications in cache replacement policies, highlighting the transition from traditional heuristic approaches to machine learning-based adaptive strategies; Section 3 explores machine learning techniques in predicting and adapting to the content popularity changes in edge networks, Section 4 discusses the role of machine learning in securing caching strategies against a variety of threats including side-channel attacks, Section 5 compares machine learning approaches to traditional approaches, and Section 6 concludes by summarizing the insights gained from the review and identifying directions for future research.

## 2 Cache replacement

**Figure 1:**
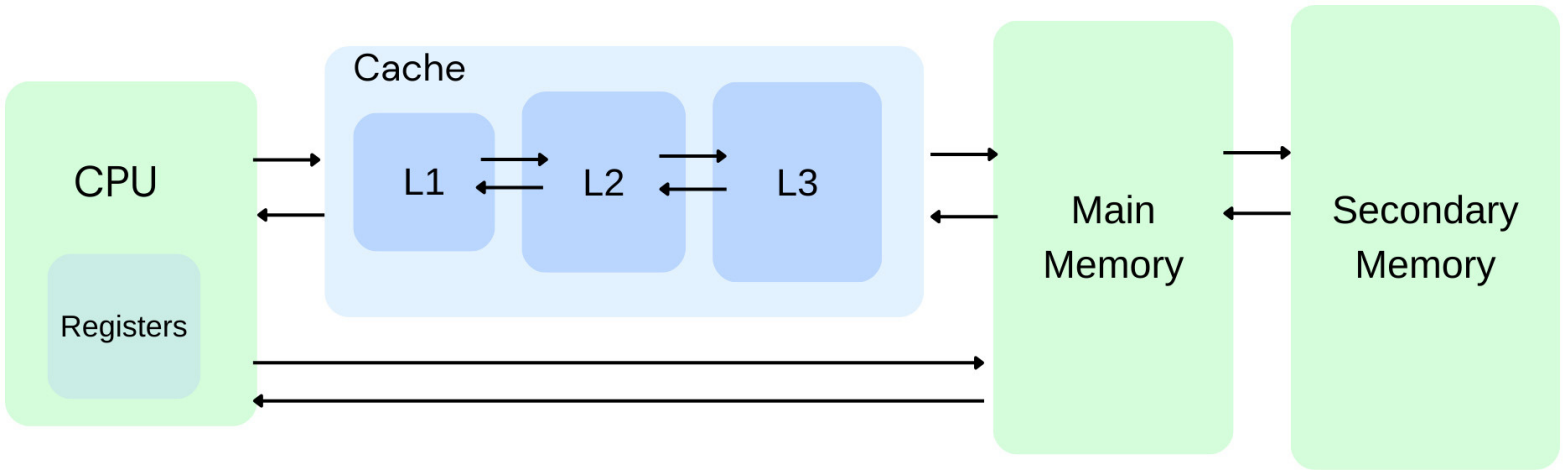
Memory block diagram for hardware caches.

**Figure 2:**
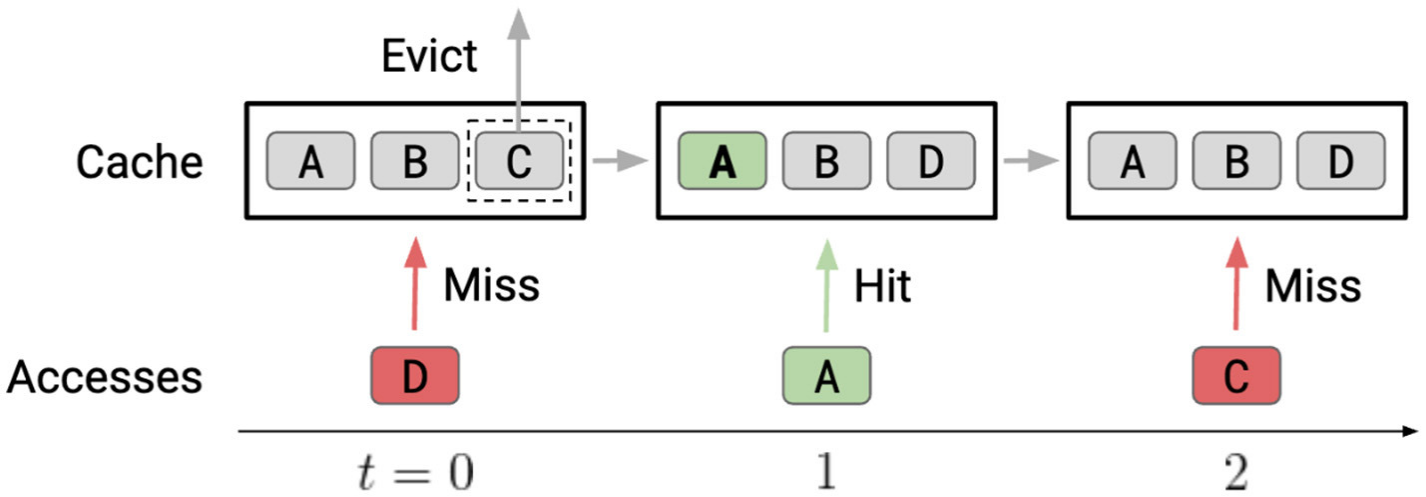
Cache replacement. At *t* = 0, line D is accessed, causing a cache miss. The replacement policy chooses between lines A, B, and C in the cache and, in this case, evicts C. At *t* = 1, line A is accessed and is already in the cache, causing a cache hit. No action from the replacement policy is needed. At *t* = 2, line C is accessed, causing another cache miss. The replacement policy could have avoided this miss by evicting a different line at *t* = 0.

Program execution speed critically depends on increasing cache hits as cache hits are orders of magnitude faster than misses since misses necessitate accessing a lower level in the memory hierarchy (Figure 1). So, cache replacement policies, which affect data kept in the cache, can critically impact performance and latency. Cache replacement policies come into play when the cache is full, and a new data block needs to be added to the cache, which necessitates the removal of a data block from memory (Figure 2). Current replacement policies typically resort to heuristics designed for specific common access patterns, which fail on more diverse and complex access patterns (Liu et al., 2020). These static policies do not have good all-around performance while having many drawbacks, like working well for some workloads but not for others (Vietri et al., 2018; Rodriguez et al., 2021).

Most commonly used policies such as least recently used (LRU) and least frequently used (LFU) suffer these drawbacks. LRU selects the least recently used to evict, while LFU chooses the least frequently used one. LRU adapts well to changing working sets but poorly handles looping patterns, whereas LFU is effective against looping patterns but struggles with changing working sets (Choi and Park, 2022).

### 2.1 Efficient cache replacement

In recent times, efficient cache management has become essential as Moore's Law has slowed down and Dennard scaling has ended (Sethumurugan et al., 2021). An efficient cache management policy is one that effectively reduces off-chip bandwidth utilization, improves overall system performance and reduces user access times while also being able to generalize to unseen code paths (i.e., sequences of accesses) from the same program as there are exponentially many code paths and encountering them all during training is infeasible (Sethumurugan et al., 2021; Liu et al., 2020; Ma et al., 2018). The following subsections discuss efficient cache management strategies.

### 2.2 Machine learning in cache replacement

Machine learning can be used in cache for a variety of things such as improving branch predictors, memory controllers, reuse prediction, prefetchers, dynamic voltage, and frequency scaling management for network-on-chip (NoC) and NoC arbitration policy (Sethumurugan et al., 2021; Shi et al., 2019).

**Figure 3:**
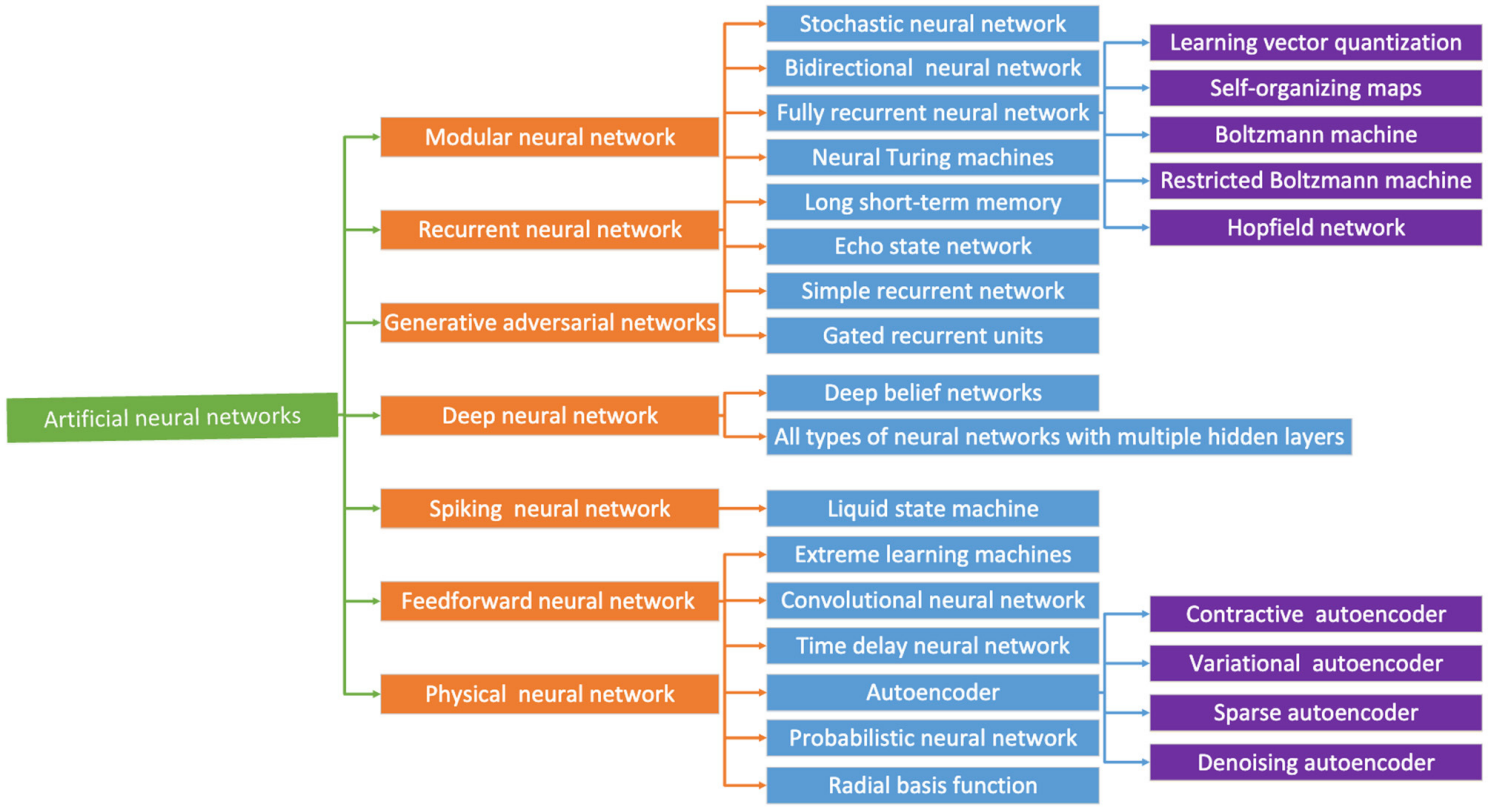
Summary of artificial neural networks (Chen et al., 2019).

Neural networks are a popular type of machine learning and can be of many types (Figure 3). However, neural networks cannot be directly implemented in hardware due to various concerns such as the power required and area and timing constraints. They require a lot of training resources and are very large and slow (in order of milliseconds) to be implemented to make predictions within nanoseconds (Sethumurugan et al., 2021; Shi et al., 2019).

**Figure 4:**
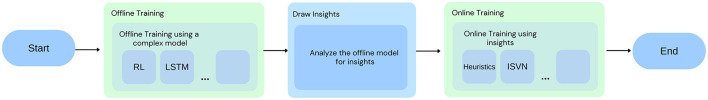
Flow of machine learning in cache replacement.

So, machine learning can be used to design an offline model and analyze the neural network for insights to derive a feasible replacement algorithm to implement in hardware (Figure 4).

Reinforcement learned replacement (RLR) (Sethumurugan et al., 2021) is one such attempt. In RLR, reinforcement learning (RL) is used to learn offline cache replacement policy. Insights such as reuse distance can be approximated by preuse distance, the chance of cache receiving a hit can be predicted by the type of previous access, heavily accessed cache lines are likely to be reaccessed in future, and recently inserted cache lines are prioritized for eviction to allow older cache lines to be reused, are drawn from analysis of the offline model. These insights are then used to design a replacement algorithm for online replacement. RLR improves both single-core and four-core system performance by 3.25 and 4.86% when compared to LRU.

A lot of existing cache replacement policies use PC information such as PC-based Re-reference Interval Prediction (PC-RRIP), PC-based Dynamic Insertion Policy (PC-DIP), and PC-based Set Dueling (PC-SD). These policies have high hardware overhead, face difficulty capturing the temporal locality, and are too sensitive to workload characteristics. RLR is exempt from these limitations while having better performance. In addition, the design of RLR is such that it can be easily integrated with existing single/multi-core systems (Sethumurugan et al., 2021).

Another such attempt is demonstrated in the study by Shi et al. (2019), in which offline training is done on an unconstrained and powerful RNN for individual programs and insights are drawn from this model to design a much simpler policy that can be easily implemented in hardware with order of magnitude lower cost and similar accuracy. It consists of three steps: (1) offline caching model using an LSTM with a scaled attention layer, (2) offline analysis for insights, and (3) an online model using IVSM. The major insight drawn was that optimal caching decisions can be predicted with a long history of past load instructions. Still, instead of depending on the full ordered sequence, it only depends on the presence of a few PCs. This insight helps Glider triumph over other machine learning approaches as it uses an unordered list of unique PCs, thereby increasing the control-flow history for the same hardware budget and training speed. An IVSM is used for online inference as it is much easier to implement in hardware. Because of its simplicity, it converges faster than an LSTM while also achieving good accuracy. The Glider cache replacement policy was evaluated on 33 memory-intensive programs from the SPEC 2006, SPEC 2017, and GAP benchmark suites. In single-core settings, Glider reduced the miss rate by 8.9% compared to LRU, outperforming other leading algorithms such as Hawkeye (7.1%), MPPPB (6.5%), and SHiP++ (7.5%). In a four-core system, Glider improved instructions per cycle (IPC) by 14.7%, surpassing Hawkeye (13.6%), MPPPB (13.2%), and SHiP++ (11.4%) (Shi et al., 2019).

Machine learning can also learn from and improve existing cache replacement strategies. Machine learning-based LeCaR (Learning Cache Replacement) (Vietri et al., 2018) is an attempt to improve Adaptive Replacement Cache (ARC), which has better performance than LRU. The Adaptive Replacement Cache (ARC) is an advanced caching algorithm designed to improve cache performance by dynamically adjusting to access patterns. It divides the cache into two main segments: one for recently accessed items and one for frequently accessed items.

LeCaR solves ARC's performance loss when a “stable” working set does not fit in the cache. It is a scalable solution as it scales well when workloads get larger relative to cache sizes. LeCaR assumes that at every instant, the workload is best handled by a judicious “mix” (i.e., a probability distribution) of only two fundamental policies: recency-based and frequency-based evictions and the weight associated with the two policies is not a function of their current hit rate but of the current associated regret. During cache miss, either LRU or LFU is chosen randomly using the probabilities derived from their associated cumulative regret values due to the misses they “caused.” The LeCaR framework outperforms ARC by over 18 times when using only two basic eviction policies, LRU and LFU, particularly when the cache size is small compared to the working set size.

CACHEUS (Rodriguez et al., 2021) is similar to LeCaR but has an advantage over LeCaR by being completely adaptive, with having no statically chosen hyper parameters such as learning rate and discount rate (fixed values of these parameters are used in LeCaR), which increases its flexibility.

Like the above, Zhou et al. (2022) proposes a framework to learn the relationship between workload characteristics and probability distribution of replacement policies. The proposed replacement algorithm, Catcher, uses deep reinforcement learning (DRL) to learn to choose LRU or LFU for replacement.

Another approach, PARROT (Liu et al., 2020), cast cache replacement as learning a policy on an episodic Markov decision process to leverage techniques from imitation learning. It tries to learn from Belady's optimal policy, which computes the theoretically optimal caching decision using knowledge of future cache access. PARROT maintains high cache hit rates even on complex programs, showing that it can generalize to new code paths not seen during training.

Apart from these frameworks, Choi and Park (2022) leverages machine learning, specifically Seq2Seq modeling based on LSTM networks, to predict future data block requests, aiming to avoid incorrect cache replacement decisions. It stores the predicted blocks in a prediction buffer while using a re-prediction process that increases the accuracy of the prediction buffer. Also, a non-access buffer is kept to determine the eviction target using the future block sequence in O(1) time complexity. It outperforms the LRU by 77%, the LFU by 65%, and the ARC by 77%. Future works can include improving prediction methods, enhancing the heuristic replacement policy, and exploring feasibility. Table 1 summarizes some of the above-mentioned approaches.

**Table 1:** Summary of cache replacement techniques in hardware caches.

**Algorithm**	**Machine learning technique used**	**Key problem with earlier approaches**	**Evaluation metric used**
Reinforcement Learned Replacement (RLR)	- Uses reinforcement learning (RL) to learn a cache replacement policy offline - Analyze the learned model to derive a new policy called Reinforcement Learned Replacement (RLR)	- Heavy Hardware Overhead with PC-based Policies - Limited Applicability of PC-based Policies due to hardware complexity and design verification overhead	System performance, Hardware overhead, Hit rate improvement
Glider cache replacement policy	- Offline, unconstrained deep RNN model - Interpret it for insights - Use insights to design a simple online model with similar accuracy to an offline model but significantly lesser cost	- Heuristic-based are customized for a limited class of known cache access patterns	Performance, Accuracy, miss rate, IPC
Machine learning-based LeCaR (Learning Cache Replacement)	- Online learning with variants of MAB - Online reinforcement learning with regret minimization	- Improves ARC: For small cache sizes, when a “stable” working set does not fit in the cache, ARC suffers a loss in performance	Hit rate, Scalability
PARROT	- Imitation learning to learn cache access patterns by leveraging Belady's	- Heuristics perform well on the specific simple access patterns but poorly on programs with more diverse and complex access patterns	Cache hit rate
Seq2Seq	- LSTM-based Seq2Seq network that can predict the next sequence of the I/O stream	- Different heuristic cache replacement algorithms suffer from different problems: LRU- poorly handles looping patterns, LFU- struggles with changing sets, combined policies lack predictive capability, resulting in suboptimal performance and potential cache pollution	Hit rate, predicting future sequences

#### 2.2.1 Comparison of reinforcement learning and supervised learning

As is evident from the above discussion, supervised learning (SL) and reinforcement learning (RL) are two of the most powerful tools for cache replacement. While SL models are trained on historical cache access data, RL models interact with the system environment and learn optimal policies over time. In terms of accuracy, since SL predicts cache replacement based on previous cache accesses, they work well when access patterns are stable. In real-world scenarios, though, workloads are dynamic, and access patterns evolve over time, leading to the problem of “concept drift.” As noted in Sethumurugan et al. (2021), SL models struggle to maintain accuracy without frequent retraining. RL models can adapt to dynamic workloads and do not require labeled training data as in SL models. It learns optimal cache replacement policies through exploration and reward feedback. Li et al. (2019) demonstrates that RL models take dynamic characteristics of workloads into consideration and work especially well in environments where access patterns change frequently. RL's ability to learn from ongoing interactions allows it to provide better cache hit rates in non-stationary workloads.

However, in terms of resource requirements, RL models, especially deep reinforcement learning (DRL), are much more resource-intensive than their SL counterparts. They require significant computational resources during training to explore and update policies. Zhou et al. (2022) noted that RL models must balance exploration and exploitation, which demands higher memory and computational power compared to SL models.

In summary, machine learning methods enhance cache hit rates by leveraging historical data to predict future requests more accurately than traditional caching algorithms. Techniques such as reinforcement learning, for instance, learn from past cache hits and misses to make better caching decisions over time. Similarly, deep learning models, such as like convolutional neural networks (CNNs), can identify complex patterns in access sequences, improving the prediction of which content to cache. Compared to traditional algorithms such as least recently used (LRU) or Least Frequently Used (LFU), which rely on simplistic heuristics, machine learning models adapt to changing access patterns, thereby optimizing cache efficiency and reducing latency.

## 3 Content caching in edge networks

Due to a rapid increase in streaming rich multimedia content like YouTube, a major challenge has emerged to maintain users' Quality of Experience (QoE) in terms of perceived latency. Caching at network edges like routers becomes essential to handle this explosive growth while maintaining the network performance and user's QoE (Narayanan et al., 2018; Tanzil et al., 2017; Shuja et al., 2021; Berger, 2018).

Information-centric networks (ICN) have been developed as an architecture for content delivery to tackle this growth and related issues. It introduces new features like in-network caching, where routers can cache objects and serve user requests directly. This caching approach reduces network load and perceived latency because it minimizes the need to fetch every request from the origin server (Narayanan et al., 2018). In-network content caching meets the demands while also helping to reduce network traffic.

**Figure 5:**
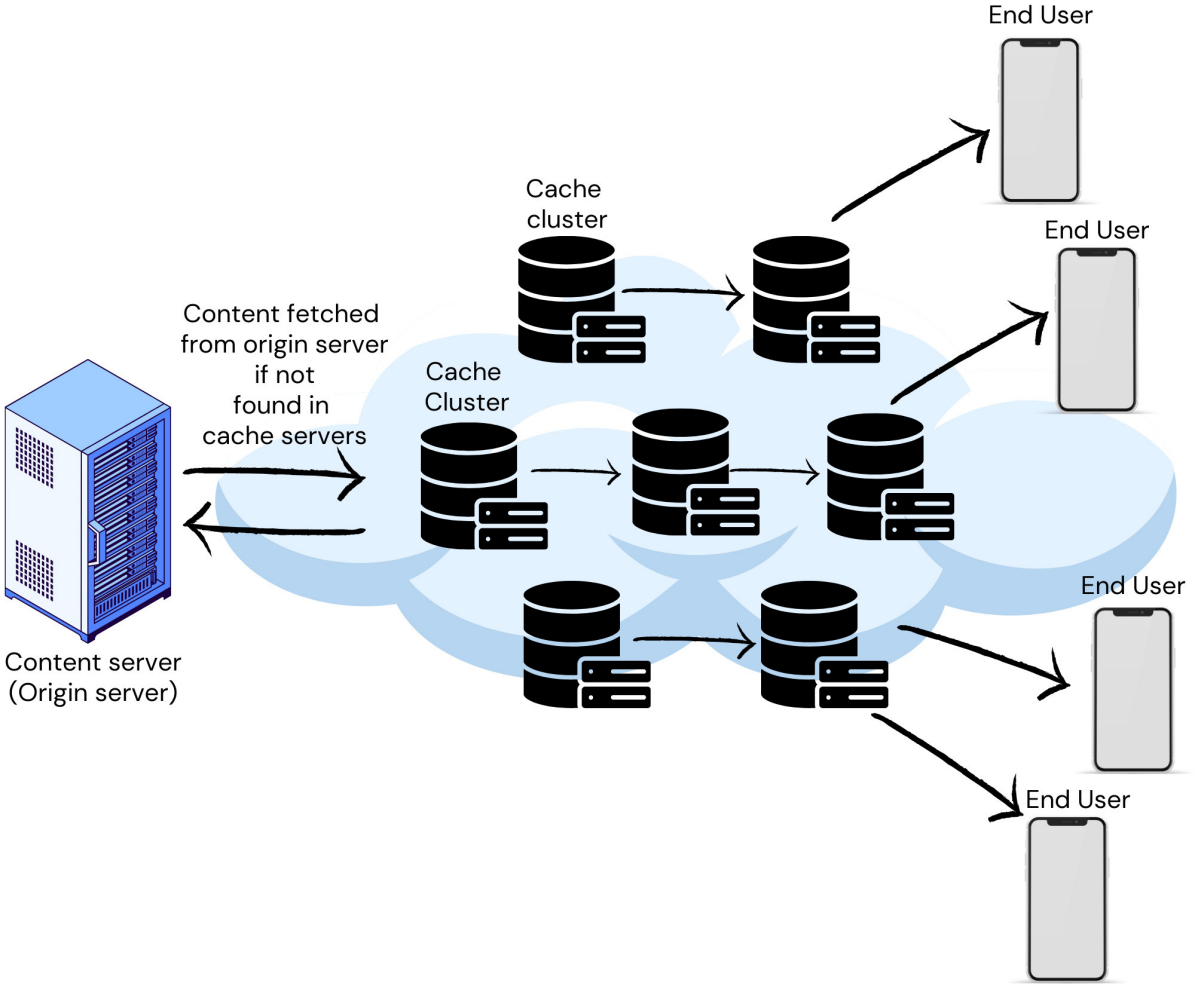
Typical content delivery network (CDN).

By storing data close to end-users, Edge Caching increases user engagement and content provider revenue while reducing latency and network congestion by minimizing redundant data traffic (Chang et al., 2018; Shuja et al., 2021; Cheng et al., 2018). Due to limited cache capacity, cache admission and eviction strategies are paramount. Limited resources and bursty requests distinguish edge caching from traditional content delivery networks (CDNs), making existing caching algorithms ineffective in handling the dynamic nature of edge network requests, necessitating the development of new caching algorithms (Fan et al., 2021). Figure 5 illustrates a typical CDN with edge caching.

### 3.1 Feature engineering

Feature engineering is a critical step in developing machine learning models for cache management, involving the selection, creation, and transformation of relevant features from raw data. Research has shown that proper feature engineering can significantly enhance model performance in cache management systems.

Transforming raw access logs into features that capture the recency and frequency of content requests is essential for predicting future cache hits. The work by Savanović et al. (2023) emphasizes the importance of recency and frequency features in identifying frequently accessed content in healthcare IoT systems. This approach allows machine learning models to prioritize the caching of content that is likely to be accessed again in the near future, improving cache hit rates and reducing latency.

Creating features that represent daily or weekly access patterns can help models anticipate recurring spikes in demand. Temporal features enable models to adjust cache policies based on known periods of high demand, such as during peak usage hours in content delivery networks.

#### 3.1.1 User segmentation

Engineering features based on user profiles or segments allow models to personalize caching strategies for different user groups.

#### 3.1.2 Content aging

Introducing features that account for the aging of content popularity over time helps models decide when to evict stale content from the cache. By tracking how long content has been in the cache without being accessed, models can make informed decisions about when to remove or replace cached data, optimizing resource use.

### 3.2 Training data for machine learning

Training machine learning models for cache management requires diverse data to accurately reflect usage patterns and predict future requests. Various researchers have outlined the key types of data that are essential for these models.

#### 3.2.1 Request frequency

How often specific content is requested is crucial for identifying popular content. Studies such as Alkanhel et al. (2023) highlight the importance of request frequency as a feature in network security applications, where frequently accessed content requires faster response times and optimal caching decisions. Similarly, Salb et al. (2023) emphasizes that request frequency is a key feature in models for improving cache performance in IoT networks.

#### 3.2.2 Temporal patterns

Time-based data, such as the time of day or week, can indicate periodic spikes in content requests. Temporal features are important for predicting peak access times and adapting caching policies accordingly. These patterns are especially useful in systems such as edge caching, where user activity can fluctuate significantly throughout the day.

#### 3.2.3 Content type

Differentiating between content types (e.g., video, text, and image) is critical for applying caching strategies suited to each content type. For example, Narayanan et al. (2018) demonstrates how models trained on content type can prioritize caching of multimedia content to minimize latency in content delivery networks. Content-specific caching ensures that high-bandwidth data, such as video, are given priority in cache management decisions.

#### 3.2.4 User behavior

Data on user navigation paths, session durations, and engagement metrics can indicate the likelihood of repeated access to certain content. Research such as Saheed et al. (2024) shows how user behavior data are used to predict content popularity in healthcare systems, which are prone to recurring access patterns based on user profiles. These data help cache systems to pre-load frequently accessed content, thereby improving cache hit rates.

#### 3.2.5 Network conditions

Information on network bandwidth, latency, and congestion is essential for optimizing caching strategies based on current network performance. Studies such as Savanović et al. (2023) and Salb et al. (2023) discuss how dynamic network conditions can affect cache management, with machine learning models adjusting cache policies to account for real-time network metrics. By integrating network condition data, caching systems can dynamically allocate resources to maintain optimal performance even under varying loads.

### 3.3 Content popularity prediction

Past caching algorithms can be classified as reactive and proactive but have limitations. Reactive caching, such as LRU, and LFU, due to no information of future content popularity, leads to caching non-popular objects and evicting them before they are accessed. Proactive caching, conversely, cannot deal with non-stationary object access patterns such as sudden changes in content popularity (Narayanan et al., 2018). Some methods rely on Zipf distribution assumption or require intrusive user information, limiting applicability (Tanzil et al., 2017).

Many machine learning models have been developed utilizing techniques such as clustering, classification, regression, and reinforcement learning to learn content demand based on user behavior, content characteristics, and temporal dynamics (Chang et al., 2018).

**Figure 6:**
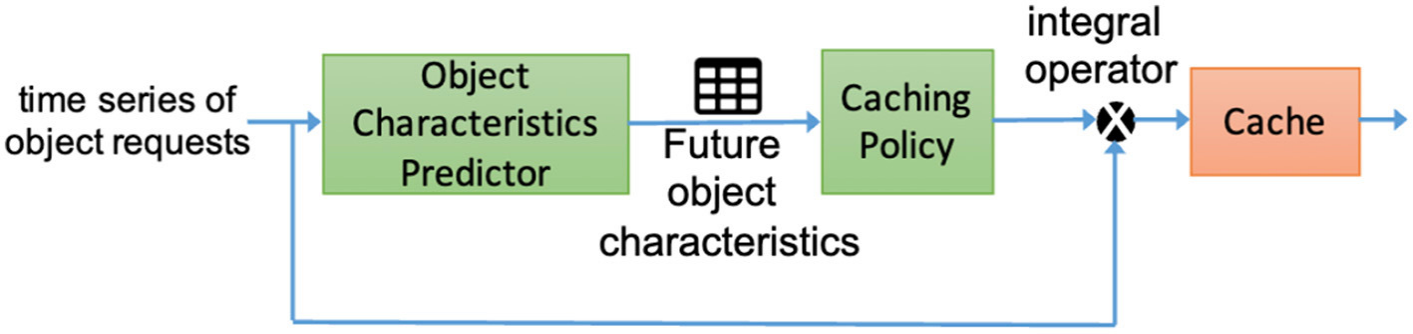
Application of DeepCache to a time series of object requests aims to predict object popularity and increase cache hit efficiency (Narayanan et al., 2018).

DeepCache (Narayanan et al., 2018) is one of the attempts to resolve these issues. DeepCache uses a deep LSTM Encoder-Decoder model to predict object characteristics. It learns the changes in request traffic patterns to predict content popularity, then, using a caching policy component, decides to cache objects or not to maximize cache hits (Figure 6).

Another approach (Tanzil et al., 2017) to calculate content popularity utilizes extreme learning machine based on users' behavior, content features, and requests statistics from users as they become available. The Mixed-Integer Linear Program (MILP) utilizes popularity estimates for cache initialization and determines what content has to be cached initially. Later, the Segmented Least Recently Used with three segments (S3LRU) using content requests decides whether to cache the content or not at a later time. Real-world YouTube data and an NS-3 simulator are used to demonstrate the effectiveness of the caching scheme.

Apart from these, Popularity Aware (PA) Cache (Fan et al., 2021) is another such framework. It learns time-varying content popularity adaptively using multilayer RNN and hedge backpropagation strategy. Unlike conventional deep neural networks (DNNs), which learn a fine-tuned but possibly outdated or biased prediction model using the entire training dataset with high computational complexity, PA-Cache weighs a large set of content features and trains the multilayer recurrent neural network from shallow to deeper when more requests arrive over time. This significantly reduces the computational cost. It consists of two phases: offline content popularity prediction and online replacement decision. Offline prediction involves forecasting content popularity within defined time intervals. Online replacement decisions utilize these predictions to make real-time cache replacement decisions.

Thar et al. (2019) proposes a caching scheme for virtualized networks to increase cache hit rates and access latency of mobile virtual network operators (MVNOs) video content, which helps reduce capital and operation costs. Using reinforcement learning, the best hyperparameters are chosen for the deep learning model, which predicts future cache demand and request count. Thar et al. (2018) uses deep learning to predict the future popularity scores of content based on their predicted class label and then caching the contents with high popularity scores.

### 3.4 Other approaches

While many approaches are based on predicting content popularity, many others use different methods. One of them (Zhong et al., 2018) uses Deep Reinforcement Learning (DRL) for content caching at edge nodes. It aims to maximize the long-term cache hit rate without requiring knowledge of content popularity distribution. It defines state and action spaces and a reward function for the DRL agent to maximize the cache hit rate while using Wolpertinger architecture within the DRL framework. Compared with such as LRU, LFU, and FIFO, this results in better short-term and stable long-term cache hit rates for DRL. It also offers comparative cache hit rates with Deep Q-learning with reduced runtime. Future research can explore DRL agents for scenarios involving multiple base stations (currently, only a single base station is considered), consider content size variations and user preferences, and extend the framework to address caching problems in device-to-device communications.

Another approach, RL-Cache (Kirilin et al., 2019), uses model-free reinforcement learning (RL) for cache admission decisions. It utilizes Direct Policy Search (DPS) type model-free machine learning, using MC sampling to search for a better policy directly in the policy space. On being evaluated on Akamai's production traces across web, image, and video traffic classes, it outperforms state-of-the-art algorithms in cache hit rate. Also, it demonstrates robustness by being trained in one location and executed in another within the same geographic region. Future work can focus on adding cache eviction to the RL-Cache algorithm.

**Figure 7:**
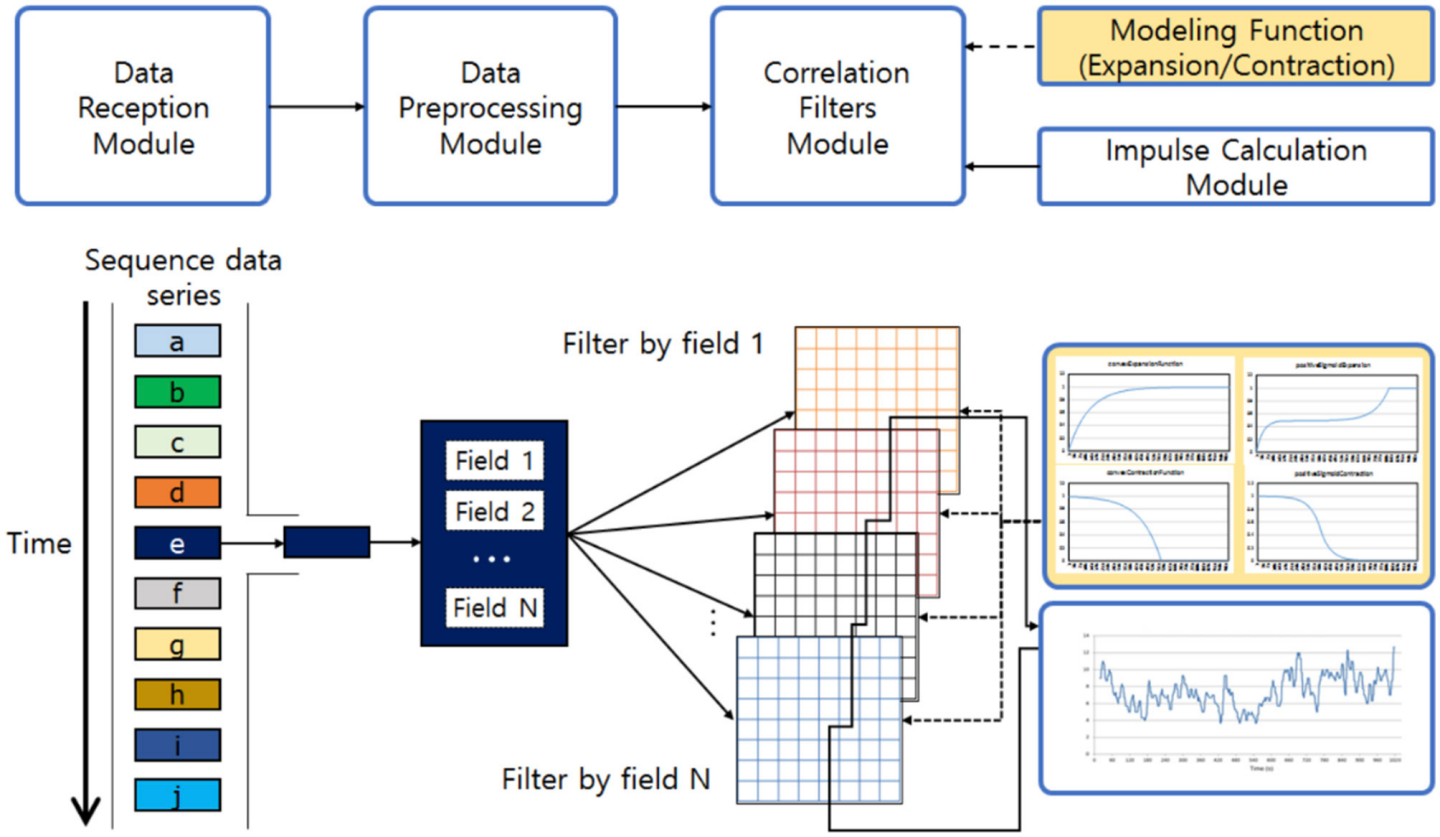
SNN architecture comprises four main modules: data reception, data preprocessing, correlation filters, and impulse calculation (Im et al., 2018).

One of the characteristics of an efficient caching algorithm is to utilize the inter-relationships between sequenced requests as there is often some correlation among them (Im et al., 2018). SNN-Cache (Im et al., 2018) is an attempt in that direction. SNN-Cache is based on stimulable neural network (SNN). This machine learning-based relation analysis system analyzes the relationship among sequenced data in real-time and with low computational complexity. SNN-cache differs from previous caching policies since earlier ones are usually based on recency, frequency, or cost. In contrast, SNN-Cache analyzes the relationships among data to make caching decisions. Real-valued matrix filters are used in SNN to capture the inter-relationships among data items. As new data enter the cache, the data names are used to pinpoint regions within the filters for updates. With each incoming request, filter values are updated, enabling consideration of structural similarities and temporal relationships among different data items. Its architecture consists of Data Reception, Data Preprocessing, Correlation Filters, and Impulse Calculation modules (Figure 7). SNN can also be used on problems such as market basket analysis and online recommendation systems, which can be taken as future work.

Caching techniques can impact energy consumption as well. Li et al. (2019) focus on energy consumption in cache-aided ultra-dense networks (CUDN) and propose a novel caching strategy. It utilizes the DRL-based Deep Q-learning network while optimizing the parameters and structure of the deep Q neural network based on the latest findings. Google TensorFlow is used for implementation with Adam Optimizer to optimize the loss function. It considers scenarios with dynamic and unknown content popularity, making estimation of popularity difficult. Simulation results show better energy efficiency and performance with existing methods. Table 2 summarizes some of the above-mentioned approaches.

**Table 2:** Summary of machine learning approaches for content caching in cloud and edge computing environments.

**Algorithm**	**Key benefits/findings**	**Machine learning approach**	**Evaluation metrics**
DeepCache	- Enhanced network performance - Utilization of request traffic for content popularity prediction	LSTM-based content popularity prediction	Cache hit rate, Network performance
Learning-based dynamic cache management in a cloud	- Improved cache hit rate while maintaining QoS - Dynamic cache allocation in the cloud leveraging learning techniques	Learning-based regression techniques [Support Vector Regression (SVR), Gaussian Process Regression (GPR), and Fully Connected Neural Network (FCN)]	Cache hit rate, QoS maintenance, Cache space optimization
SNN-Cache(Stimulable Neural Network)	- Reduced traffic load over the network - Utilization of inter-relationships for caching decisions	Relation analysis by using a set of real-valued matrix filters	Content server load reduction, Cache utilization
Adaptive Scheme for Caching	- Enhanced user QoE and network performance - Adaptive caching based on predicted content popularity	User behavior analysis using extreme learning machine (ELM)	User QoE improvement, Network performance enhancement
Deep RL-Based Content Caching	- Improved cache hit rates compared to traditional algorithms - Competitive performance with deep Q-network, with reduced runtime	Deep Reinforcement learning with Wolpertinger architecture	Cache hit rate, Runtime efficiency
Cache Strategy for Cache-Aided Ultra-Dense Network	- Enhanced Content retrieval efficiency and network throughput through decreased duplicate content transmissions - Improved energy efficiency in Cache-Aided Ultra-Dense Networks	Online learning algorithm for content placement based on DRL using deep Q neural network	Content retrieval efficiency, Network throughput, Energy efficiency
RL-Cache	- Improved hit rates compared to state-of-the-art methods - Modest resource overhead on CDN servers - Robust and portable training across different locations within the same geographic region - Easier to implement as a front end for existing CDN caches.	Model-free RL—Direct Policy Search (DPS) algorithm used	Hit rates, Resource overhead, Portability
PA-cache	- Significantly reduces computational cost compared to conventional deep neural networks based approaches - Outperforms existing caching algorithms in real-world scenarios	Evolving multi layer recurrent neural network (RNN) architecture using hedge backpropagation strategy	Hit rates, Computational cost, Real-world performance

## 4 Cache security

Caches are used extensively in modern processors to have low latency by skipping access to the main memory. Cache side-channel attacks (CSA) target shared hardware resources to retrieve sensitive information from victims sharing the resource (Tong et al., 2020). Cache side-channel attacks can be Flush + Reload, Flush + Flush, Prime + Probe, or Specter Attack (Sayadi et al., 2020; Depoix and Altmeyer, 2018; Tong et al., 2020).

**Figure 8:**
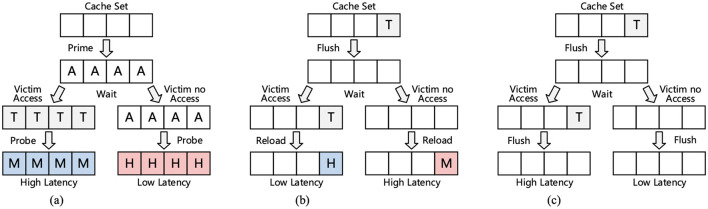
Illustrations of **(A)** Prime + Probe, **(B)** Flush + Reload, and **(C)** Flush + Flush attacks. Here, T is target data, H is a cache hit, M is a cache miss, and A is attacker's data (Shen et al., 2021).

Figure 8 illustrates various cache side-channel attacks. In Flush-Reload, the attacker flushes the data from the cache, and waits for the victim to execute, and then data are reloaded by the attacker by accessing it. If the time for reload is shorter, the attacker knows that the victim accessed the data. Flush-Flush is similar to Flush-Reload, except the attacker flushes instead of reloading the shared memory blocks. Longer time taken for flushing indicates data access by the victim. Prime + Probe attacks target L1/L3 caches and have two stages: Prime, in which the attacker creates an eviction set and fills the cache with it, and a probe stage, in which the attacker re-accesses the eviction set after victim execution. A longer access time indicates victim data access. Specter attacks exploit speculative execution by the CPU by tricking it into executing instruction sequences that leak information (Sayadi et al., 2020).

To counter the inefficiency of traditional software-based detection methods, most processors, such as Intel, ARM, and AMD, come built with hardware performance counters (HPCs) to track low-level hardware events for security risk detection and vulnerability checks. HPCs are registers built-in processors to capture and monitor hardware events such as cache memory accesses and misses, TLB hits and misses, and branch mispredictions (Tong et al., 2020; Depoix and Altmeyer, 2018; Sayadi et al., 2020; Mushtaq et al., 2018a,b).

### 4.1 Machine learning in cache security

Machine learning algorithms have the advantage over traditional approaches in that they have decreased latency in attack detection and reduced hardware/resource utilization overheads (Sayadi et al., 2020). Furthermore, traditional approaches cannot detect complex unknown attacks as they depend on static signature analysis of applications executed (Sayadi et al., 2020). Unlike traditional heuristics-based policies, previous research proves that a lot fewer false positives are created by neural networks, which helps reduce unnecessary killings of processes (Depoix and Altmeyer, 2018).

**Figure 9:**
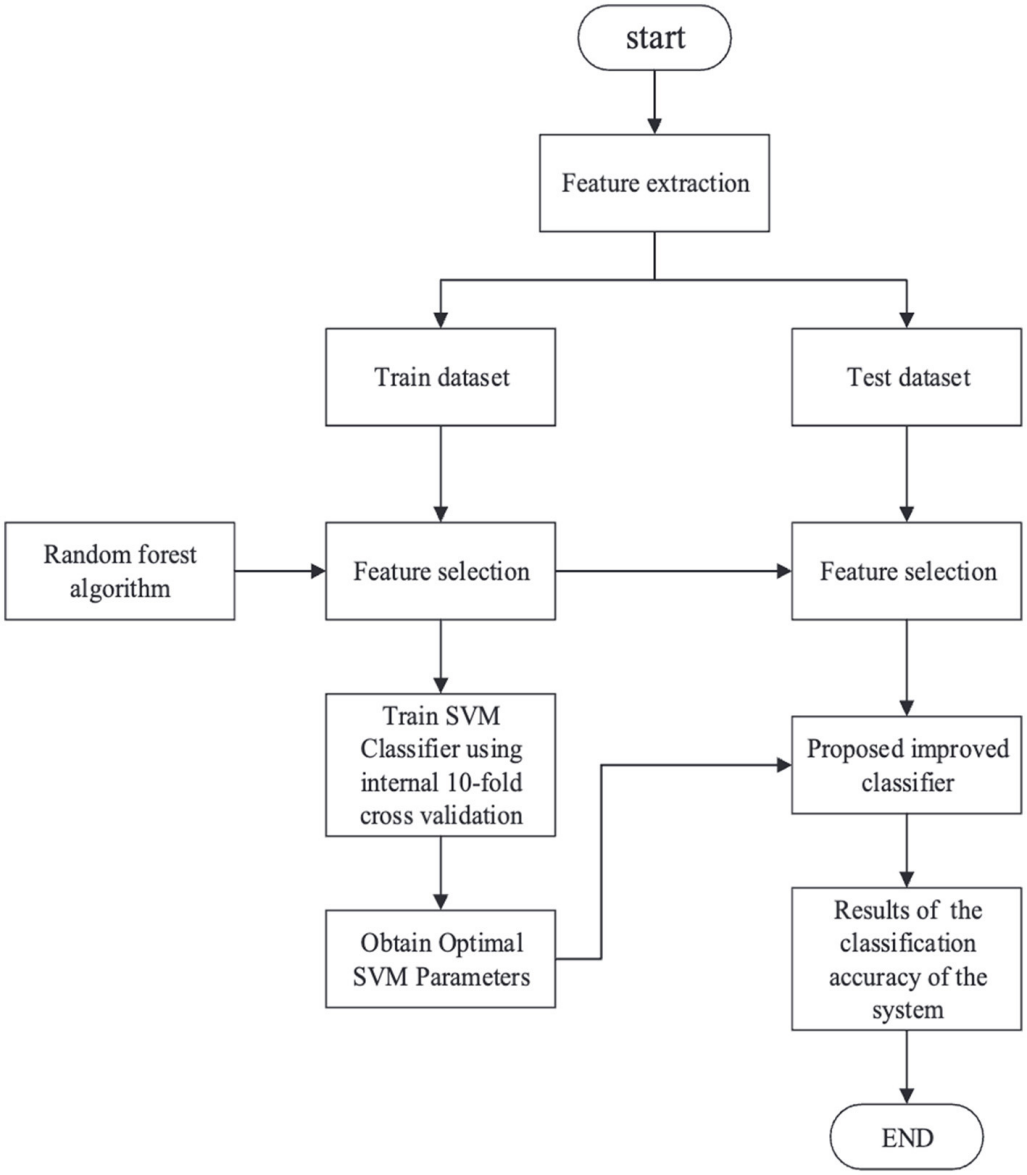
Step-by-step process for detecting cache-based side-channel attacks using machine learning algorithms (Tong et al., 2020).

Previous works on CSA detection are highly specialized for an individual attack type with high detection overhead. To handle this limitation, Tong et al. (2020) attempts to establish a unified detection model for Flush + Reload, Prime + Probe, and Flush + Flush attacks using minimal hardware events while maintaining high accuracy in detecting CSAs based on the AES algorithm. It uses Hardware performance counters to gather hardware counter features under different attacks and then uses a random forest algorithm to filter those features into only four features. SVM is later used to model the system. The machine learning algorithm mainly consists of five steps: data extraction, data division, model training, model testing, and classification results (Figure 9). On being evaluated using SPEC benchmark tests, the model exhibits high accuracy rates of 99.92%, 99.85%, and 96.58% for attacks under no load, average load, and full load conditions, respectively. Future work can focus on the actual implementation of the model in hardware, and more analysis can be done during actual use, as well as on the overhead of adding it to the system.

Another method (Depoix and Altmeyer, 2018) focuses on real-time detection of Specter attacks. While existing approaches tend to prevent speculative execution to mitigate this attack, it can lead to serious performance issues. Depoix and Altmeyer (2018) overcomes this limitation by not limiting speculative execution but training a neural network on a dataset of HPC data for malicious and benign processes to detect malicious activities. Three processor events (L3 cache misses, L3 cache accesses, and total instructions) are selected to characterize Specter attacks. The neural network architecture consists of three input neurons for the HPC data for the events and sigmoid output for classification. The neural network achieves over 99% accuracy in detecting Specter attacks in the test environment.

**Figure 10:**
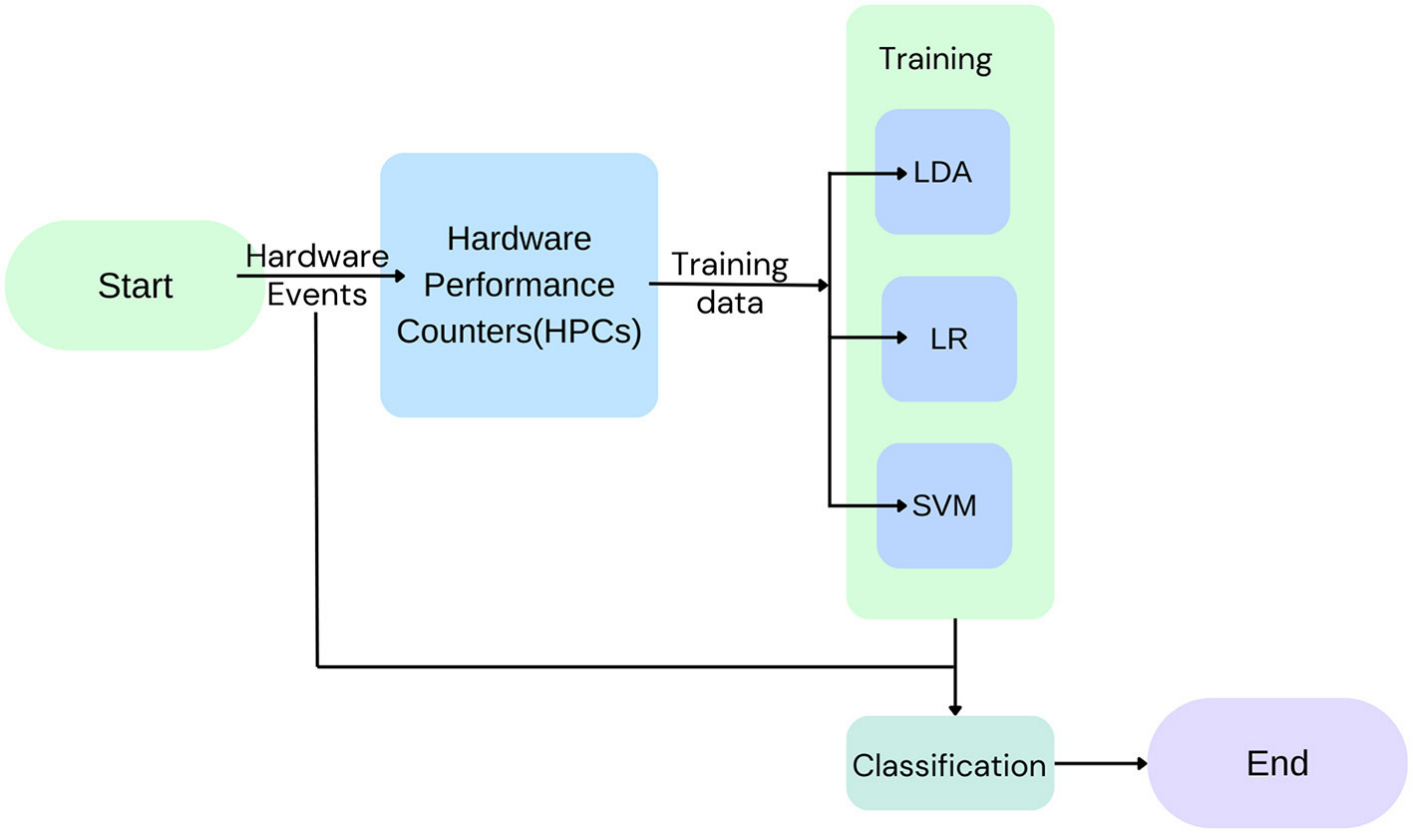
Low-level hardware events are recorded in HPCs and used to train the three models, linear discriminant analysis (LDA), logistic regression (LR), and support vector machine (SVM), which are used for the classification of events as attacks or not.

Mushtaq et al. (2018b) uses three machine learning models, namely, linear discriminant analysis (LDA), logistic regression (LR), and support vector machine (SVM), for run time detection of cache-based SCAs on RSA and AES crypto-systems (Figure 10). On being evaluated under different load conditions, for Flush + Reload attacks, accuracies of 99.51%, 99.50%, and 99.44% in the case of NL, AL, and FL conditions and for Flush + Flush, accuracies of 99.97%, 98.74% and 95.20% for NL, AL, and FL conditions were obtained. Future works could focus on integrating more machine learning models and applying the detection module to more CSAs.

Mushtaq et al. (2018a) provides quantitative and qualitative analysis of 12 machine learning models, six linear and six non-linear, in detecting cache side-channel attacks. It compares them on classification accuracy, implementation feasibility, and overhead. Gulmezoglu et al. (2017) proposes a machine learning-based technique to detect and classify applications based on their cache access profiles in a cloud environment. It uses feature vectors to train models using support vector machines, which can classify the applications with a high degree of success. This classification can be used to discover a vulnerable application, thus protecting cloud infrastructures from cross-VM attacks.

In another study, Salb et al. (2023) explored the use of a hybrid CNN and XGBoost model, optimized via the Modified Reptile Search Algorithm, to enhance the security of IoT networks. This model improved detection accuracy by learning complex patterns in the data, which can also be applied to secure cache management systems by identifying anomalies in cache access patterns.

Similarly, Alkanhel et al. (2023) proposed a network intrusion detection system based on feature selection combined with hybrid metaheuristic optimization. This method effectively reduced false positive rates and improved detection accuracy, indicating that similar approaches could be adapted for cache security, where identifying malicious access patterns is crucial.

Savanović et al. (2023) introduced a metaheuristic-optimized machine learning framework for intrusion detection in Healthcare 4.0 IoT systems. The use of optimization techniques enhanced the model's ability to detect complex, evolving threats. Integrating such frameworks into cache management systems could bolster their resilience against sophisticated side-channel attacks and other security vulnerabilities.

In another study, Saheed et al. (2024) developed a hybrid fusion model using the Bat Algorithm and Residue Number System for feature selection in intrusion detection systems. This innovative approach reduced the computational burden while maintaining high detection accuracy, which is essential for real-time cache security in environments where both performance and security are critical.

However, a major limitation of these machine learning-based anomaly detection systems that work by identifying unusual access patterns is the presence of false positives. Anomaly detection models treat any deviation from the norm as suspicious, leading to the misclassification of legitimate access events. Tong et al. (2020) and Mushtaq et al. (2018b) demonstrate that while machine learning methods such as LDA and logistic regression-based methods achieve high detection rates, they often mistake legitimate accesses as attacks under full load (FL) conditions. This results in system inefficiencies, since important processes are killed, and unnecessary alerts. But various approaches are aware of this problem and use different means to handle them, such as combining many different methods (for example, Chiappetta et al., 2016) or checking if suspicious behavior persists over some continuous intervals (Wang et al., 2020), which are discussed later.

Concept drift causes the handling of false positives to get tricky. Concept drift refers to the changes in cache access patterns over time. Models trained on historical data may not generalize to future patterns, reducing their detection accuracy. Tong et al. (2020) proposed adaptive machine learning models that monitor hardware performance counters (HPCs) and update models to handle concept drift. Without such updates, models may misclassify legitimate accesses as anomalies.

Chiappetta et al. (2016) does detection of cache-based side-channel attacks using three primary methods: correlation-based, anomaly detection, and supervised learning. The correlation-based approach identifies similarities in L3 cache access patterns between a spy and a victim since, essentially, they are similar as they perform operations in a loop while accessing fixed memory addresses. The confidence of correlation was obtained between spy and victim ranging from 0.095 to 5.4 for AES benchmark spy and victim and 0.0016–1.66 for ECDSA benchmark spy and victim. In contrast, benign processes such as Apache webserver exhibit maximum confidence values below 0.000566, minimizing false positives. The latter two approaches use machine learning techniques since a small number of false positives may come up with the correlation-based approach. Anomaly detection treats known spy behavior as normal and flags deviations as anomalies. Using features such as L3 cache misses, total CPU cycles, and L3 accesses, it achieved an *F*-score of 0.51 for AES and 1.0 for ECDSA, with prediction times of 0.2 ms for 100 samples.

Supervised learning, specifically using neural networks, classifies cache processes as benign or malicious. It achieved *F*-scores of 0.93 for AES and 1.0 for ECDSA, with predictions taking 0.64 ms for 100 samples. This approach offers high accuracy but requires computational overhead for training. The combined use of these methods ensures comprehensive detection, balancing detection speed, computational overhead, and false positive rates, making them robust against side-channel attacks like FLUSH + RELOAD.

Some approaches offer a hybrid detection and mitigation structure. Hybrid-Shield (Wang et al., 2020) is one such framework for runtime defense against cache-based side-channel attacks (SCAs) such as Flush + Reload and Prime + Probe. The detection system utilizes hardware performance counters (HPCs) and machine learning classifiers to identify SCAs in real-time. Key features from HPCs, such as L1 hits, L3 misses, and branch mispredictions, are transformed into feature vectors, and dimensionality reduction techniques select the most impactful HPCs. Classification models, including OneR, J48, BayesNet, and Multilayer Perceptron (MLP), achieve high detection accuracy with low false alarm rates. To overcome the limitations of previous approaches of high false alarm rates, the False Alarm Minimization (FAM) technique is employed, where the “under attack” decision is delayed until some continuous intervals (delayed number, DN) report attack-like situations. This caused the false alarm rate of J48 dropped from 13.6 to 1.7% with a delayed interval (DN) of 4. The detection process is both accurate and fast, with 100% of attacks detected within 20 intervals (10 ms) for OneR and Decision Tree (DT) models.

To mitigate SCAs, Hybrid-Shield employs a cross-layer strategy that dynamically adjusts CPU frequency (1,600–3,200 MHz) and prefetcher settings. Randomization disrupts the cache access patterns used by attackers to infer victim data. Six randomization scenarios are analyzed, where the hybrid approach (both frequency and prefetcher adjustments) offers superior protection. For mitigation evaluation, protection levels are measured by the number of bits leaked in Flush + Reload and Prime + Probe attacks. When using the Hybrid-Shield strategy, the error rates for Flush + Reload and Prime + Probe are 35% and 38%, respectively, with performance overheads of 17% and 15%, highlighting the effectiveness of cross-layer randomization in balancing protection and performance.

These studies underscore the growing role of machine learning in securing cache systems. Techniques such as hybrid models, metaheuristic optimization, and feature selection have proven effective in intrusion detection, and their application in cache security offers promising avenues for enhancing the robustness of cache management systems.

## 5 Comparison with traditional approaches

Machine learning-based cache management methods differ significantly from traditional approaches such as LRU and LFU in several key ways. Traditional methods such as LRU and LFU operate on fixed rules that do not adapt to changing access patterns in real-time. For example, LRU evicts the least recently used item, while LFU evicts the least frequently used item, both based on predefined heuristics. These methods often perform suboptimally when access patterns change abruptly or involve complex workloads. On the other hand, machine learning models such as Learning Cache Replacement (LeCaR) (Vietri et al., 2018) and Reinforcement Learned Replacement (RLR) (Sethumurugan et al., 2021) learn from historical data and can dynamically adjust to evolving workloads. By predicting future access patterns, these models improve cache hit rates over time, offering better adaptability than static approaches.

While traditional algorithms such as LRU and LFU are simple, requiring minimal computational resources, they lack the complexity needed to handle dynamic, real-world cache access patterns effectively. In contrast, machine learning-based models, such as Glider (Shi et al., 2019), use advanced techniques such as recurrent neural networks (RNNs) to model complex access patterns but require significantly more computational resources for training and implementation. This added complexity leads to higher system overheads, particularly in systems with limited processing capabilities.

Machine learning approaches, such as the Seq2Seq model (Choi and Park, 2022), significantly outperform traditional methods by predicting future cache accesses based on long-term historical trends. Unlike LRU and LFU, which react to past cache accesses, machine learning models proactively cache content by forecasting future requests. This predictive capability is particularly beneficial in environments with highly variable or bursty access patterns, such as content delivery networks (CDNs) and edge computing (Narayanan et al., 2018).

Traditional caching algorithms such as LRU and LFU are not designed to handle security challenges, such as cache side-channel attacks. Machine learning-based methods offer a unique advantage by integrating security considerations into caching decisions. For instance, models such as the hybrid CNN and XGBoost used in IoT security (Savanović et al., 2023) provide both performance and security benefits by detecting potential threats while optimizing caching. This dual capability is absent in traditional caching algorithms, making machine learning methods more suitable for modern systems where security is a concern.

## 6 Conclusion and discussion

This study explores machine learning techniques for cache replacement, which is a rising and promising avenue to improve system performance while reducing latency in modern computing environments. The study also discusses recent approaches leveraging machine learning for content caching in edge networks, which has become essential due to the exponential growth of online multimedia content and user demand for high-quality experiences. Finally, cache security is discussed, particularly cache side-channel attack (CSA) detection, and how machine learning techniques detect complex and unknown attacks with lesser latency and overhead than traditional software-based approaches.

Current research on integrating machine learning in cache management demonstrated that machine learning-based approaches such as Reinforcement Learned Replacement (RLR), Glider, LeCaR, CACHEUS, PARROT, and others for cache replacement in computer hardware and models such as DeepCache, PA-Cache, RL-Cache, and others for content caching in distributed networks are viable alternatives to traditional static caching policies such as LRU and LFU. The major advantage of these machine learning algorithms is their ability to learn and adapt to diverse and complex access patterns by leveraging real-time data and user behavior patterns. Machine learning algorithms for cache security utilize hardware performance counters to monitor low-level hardware events and offer significantly lower false positives, which reduces disruptions to legitimate processes.

However, several challenges still prevent these algorithms from being deployed practically. Some algorithms suffer from scalability and generalizability concerns as they are ineffective across all workloads, content distributions, and diverse system configurations. Integrating ML-based policies in real-world hardware environments requires careful consideration of resource constraints, power efficiency, and implementation complexity, especially in resource-constrained edge devices. Challenges include

- Computational overhead: machine learning models often require significant computational resources, which can be a barrier for systems with limited processing power or energy constraints.
- Compatibility: existing cache systems are usually optimized for traditional algorithms; integrating machine learning models requires ensuring compatibility and often re-engineering the infrastructure.
- Real-time processing: cache management requires real-time decision-making, and machine learning models must be optimized for low-latency processing to avoid becoming a bottleneck.
- Data availability and quality: machine learning models depend on high-quality, representative data. In many cases, sufficient historical data might not be available, or data may be biased, affecting model performance.

Other limitations include:

- Overfitting: machine learning models, especially those with high complexity such as deep learning, can overfit to training data, failing to generalize to unseen scenarios.
- Data dependency: these models require large amounts of data for training. In cases where data are scarce or not representative, model performance can degrade significantly.
- Bias and fairness: machine learning models can inadvertently learn biases present in the training data, leading to unfair caching decisions that might prioritize certain content or users over others.
- Interpretability: many machine learning models, particularly deep learning ones, act as black boxes, making it difficult to understand the decision-making process, which can be a drawback when debugging or optimizing cache performance.

Future research could focus on enhancing prediction (caching) and detection (security) accuracy and real-time adaptability of caching and security systems, exploring new architectures that would easily integrate with cache management hardware and investigating the feasibility of deploying these techniques at scale. In edge and distributed networks, diverse machine learning algorithms can be explored to handle dynamic content sizes, user behavior, and various network topologies. The energy efficiency of these machine learning algorithms is also an area that can be explored in future research.

All in all, by addressing the challenges of scalability, hardware implementation, adaptability, and efficiency, machine learning canin provide substantial improvements in system performance and latency and also significantly increase the resilience of modern computing systems against cache side-channel attacks and ensure security in complex computing environments, all while being efficient in energy and resources.
